# Effect of *Acinetobacter* sp on Metalaxyl Degradation and Metabolite Profile of Potato Seedlings (*Solanum tuberosum* L.) Alpha Variety

**DOI:** 10.1371/journal.pone.0031221

**Published:** 2012-02-17

**Authors:** Fabiola G. Zuno-Floriano, Marion G. Miller, Maria L. Aldana-Madrid, Matt J. Hengel, Nilesh W. Gaikwad, Vladimir Tolstikov, Ana G. Contreras-Cortés

**Affiliations:** 1 Department of Environmental Toxicology, University of California Davis, Davis, California, United States of America; 2 Departamento de Investigación y Posgrado en Alimentos, Universidad de Sonora, Sonora, México; 3 Departamento de Biotecnología, Centro Interdisciplinario de Investigación para el Desarrollo Integral Regional, Michoacán, México; 4 Department of Nutrition, University of California Davis, Davis, California, United States of America; 5 Genome Center, University of California Davis, Davis, California, United States of America; Max Planck Institute for Chemical Ecology, Germany

## Abstract

One of the most serious diseases in potato cultivars is caused by the pathogen *Phytophthora infestans*, which affects leaves, stems and tubers. Metalaxyl is a fungicide that protects potato plants from *Phytophthora infestans*. In Mexico, farmers apply metalaxyl 35 times during the cycle of potato production and the last application is typically 15 days before harvest. There are no records related to the presence of metalaxyl in potato tubers in Mexico. In the present study, we evaluated the effect of *Acinetobacter* sp on metalaxyl degradation in potato seedlings. The effect of bacteria and metalaxyl on the growth of potato seedlings was also evaluated. A metabolite profile analysis was conducted to determine potential molecular biomarkers produced by potato seedlings in the presence of *Acinetobacter* sp and metalaxyl. Metalaxyl did not affect the growth of potato seedlings. However, *Acinetobacter* sp strongly affected the growth of inoculated seedlings, as confirmed by plant length and plant fresh weights which were lower in inoculated potato seedlings (40% and 27%, respectively) compared to the controls. *Acinetobacter* sp also affected root formation. Inoculated potato seedlings showed a decrease in root formation compared to the controls. LC-MS/MS analysis of metalaxyl residues in potato seedlings suggests that *Acinetobacter* sp did not degrade metalaxyl. GC–TOF–MS platform was used in metabolic profiling studies. Statistical data analysis and metabolic pathway analysis allowed suggesting the alteration of metabolic pathways by both *Acinetobacter* sp infection and metalaxyl treatment. Several hundred metabolites were detected, 137 metabolites were identified and 15 metabolic markers were suggested based on statistical change significance found with PLS-DA analysis. These results are important for better understanding the interactions of putative endophytic bacteria and pesticides on plants and their possible effects on plant metabolism.

## Introduction

Potato is a food with the fourth highest consumption in the world. The annual production is approximately 320 million tons, and it continues to increase when compared to the production of maize, wheat and rice [1]. Potato production is affected by different diseases caused by fungi, bacteria, viruses, nematodes and insects [1], [2]. One of the most serious diseases in potato cultivars is caused by the pathogen *Phytophthora infestans* (late blight). It affects leaves, stems and tubers and disperses rapidly when climatic conditions in the field are favorable (100% humidity and 12–15°C) [3], [4]. Chemical control with pesticides such as metalaxyl is an effective method against late blight [5]. Metalaxyl [(*R,S*)-methyl-*N*-(2-methoxyacetyl)-*N*-(2,6-xylyl)-d,l-alaninate] is an important acylanilide fungicide first manufactured by the Ciba-Geigy Corporation in 1977. It is a systemic, apoplastically transported fungicide that is highly active against fungi of the order Peronosporales, by selectively interfering with the synthesis of ribosomal RNA. Metalaxyl is photostable and resistant to heat. Due to its low vapor pressure (3.3 mPa at 25°C), it is very stable in water within a pH range of 1.0 to 8.5 [6]. In soil, its lifetime is from 5 to 35 days and almost all residues are found in the first 10 cm of soil [7]. Amounts of metalaxyl applied in fields vary in different countries. For example, Mexican farmers apply 1.2 kg/ha and farmers in Belgium apply 0.3 kg/ha [8]. Around 15 applications might be necessary during the growing season but in some cases farmers exceed this number of applications. In Mexico, however, farmers typically apply metalaxyl from 33 to 35 times, with the last application 14 days before harvest [9]. This represents a risk to consumers. Although Metalaxyl does not affect reproduction in animals and is not a teratogenic or mutagenic compound, this fungicide provokes cell alterations in mouse liver at 2.5 mg kg^−1^ day^−1^. In dogs at 0.8 mg kg^−1^ day^−1^, it alters alkaline phosphatase levels in blood and causes an increase in liver and brain weight. The LD_50_ of metalaxyl in mice is 669 mg kg^−1^ (oral) and >3100 mg kg^−1^ (subcutaneous) [10]. Metalaxyl has been detected in several agricultural products (grape, tomato, potato, onion, lettuce, sunflower seeds, spinach, etc.) in countries such as New Zealand, United States, Spain, France, Italy, Germany, Brazil and Belgium [7]. In Mexico, there are no records related to the residues of metalaxyl in vegetables and fruits.

In plants, metalaxyl is taken up by roots, translocated, and extensively metabolized [11], [12], [13], [14]. Metabolism involves oxidation of a ring-methyl group, hydrolysis of the methyl ester and methyl ether bonds, and ultimately conjugation to glucose. Ring-methyl hydroxylation was found predominantly in cell suspension cultures of lettuce and grapevine [14]. Other prominent products arise from O-dealkylation, ester hydrolysis and ester hydrolysis of O-dealkylated product. The principal metalaxyl metabolite found is the acid metabolite (*N*-(2,6-dimethylphenyl)-*N*-(methoxyacetyl) alanine) [15].

There are several studies related to the use of endophytic bacteria that degrade toxic organic compounds such as pesticides in the environment [16], [17], [18]. Plant-associated endophytic bacteria and rhizospheric bacteria have been shown to biodegrade toxic organic compounds in contaminated soil and can promote phytoremediation [17]. Almost all 300,000 plant species identified contain at least one species of endophytic bacteria [19], [20], [21], [22]. Germaine et al. [18] reported that when pea plants (*Pisum sativum*) were inoculated with *Pseudomonas* endophytes that were isolated from hybrid poplars *P. trichocarpa X P. deltoids cv. Hoogvorst* capable of degrading 2,4-D, the pea plant showed no accumulation of 2,4-D and showed little or no signs of phytotoxicity when compared to inoculated controls. McGuinness et al. [16] inoculated pea plants with a bacterium expressing a specific bacterial glutathione-S-transferase (GST) isolated from *Burkholderia xenovorans* LB400, BphKLB400, wild type and mutant (Ala 180Pro). This bacterium was capable of dehalogenating toxic chlorinated organic pesticides such as chloromequat chloride. Bailey and Coffey [23] showed that eight strains of fungi and six strains of bacteria degraded metalaxyl in liquid medium. The objective of the present study was to evaluate the ability of a bacterial strain isolated from the rhizosphere of potato plants cultivated in commercial fields to degrade metalaxyl in potato seedlings alpha variety cultured *in vitro*, and identify potential metabolic biomarkers and disturbed metabolic pathways emerging in response to impact of *Acinetobacter* sp and metalaxyl on potato seedling.

## Materials and Methods

All solvents were HPLC grade and were obtained from either Fisher Scientific (USA) or JT Baker (USA). Metalaxyl (99.5% purity), *N*-Methyl-*N* (trimethylsily) trifluoroacetamide, Murashige and Skoog medium, potato dextrose agar and tissue culture agar were from Sigma-Aldrich-Fluka (SAF, Deisenhofen, Germany). Metalaxyl acid metabolite (97.4% purity) was from Syngenta (USA). Stable isotope reference metabolites ([^13^C_12_]-sucrose, [^13^C_6_]-glucose, glycerol-*d*_8_, ethanolamine-*d*_4_, ethylene-*d*_6_ glycol, aspartate *d*_3_, [^13^C_5_]-glutamate, alanine-*d*_4_, valine-*d*_8_, leucine-*d*_3_ and benzoic-*d*_5_ acid) were obtained from Campro Scientific (Emmerich, Germany). The water used was produced in-house using a Milli-Q water system (resistivity 18.2 megaohm-cm).

### Plants

Potato seedlings (*Solanum tuberosum* L. - Alpha variety) were obtained from Summit Plants Laboratories Inc. (USA). The plants were obtained at 21 days of age and were housed in a growth chamber under a photoperiod of 16 h light (184 µmol/m^2^ of active photosynthetic radiation) and 8 h dark at a temperature of 19°C and 16°C, respectively.

### Isolation of rizhospheric bacteria

Samples for bacterial isolation were collected from potato fields of Zuckerman Family Farms, Inc. in Stockton, CA (USA). All necessary permits were obtained from Mr. Kenneth N. Jochimsen, Vice President of Zuckerman Family Farms, Inc. Fields were selected based on the background of treatments with metalaxyl (3 to 10 applications in 2007 and 2008). Potato plants were extracted with part of the rizhospheric soil. Under aseptic conditions using a sterile sponge, part of the rizhospheric soil was removed using the stamping technique [24] and impressions were made with the sponge over the surface of Petri dishes containing a minimal media with metalaxyl (50 mg kg^−1^ of media). Minimal media (pH 7.0) used in the study contained 2 g (NH_4_)_2_SO_4_, 4 g KH_2_PO_4_, 6 g Na_2_HPO_4_, 0.2 g MgSO_4_-7H_2_O, 1 mg FeSO_4_-7H_2_O, 10 µg B(H_3_BO_3_), 10 µg Mn(MnSO_4_), 70 µg Zn(ZnSO_4_), 30 µg Cu(CuSO_4_), 10 µg Mo(Mo_3_). The petri dishes were incubated at 30°C for 24 h. Bacterial colonies were selected and purified using a streaked plate technique [25]. Once the bacterial strains were purified, only one strain was selected based on its growth on minimal media that was fortified with metalaxyl.

### Degradation of metalaxyl

All biodegradation experiments were carried out aerobically. Liquid minimal media (55 mL) containing 50 mg kg^−1^ of metalaxyl was inoculated with the selected bacteria (7.8×10^6^±1.643 CFU mL^−1^) and incubated at 30°C in a bath (Versa Bath, Fisher Scientific, USA) with horizontal agitation at 150 rpm for a period of 75 days. To determine if the bacteria degrades metalaxyl, two controls were included. One control consisted of liquid minimal media with metalaxyl and no bacteria added, while the other control included liquid minimal media with bacteria and no metalaxyl. Treatment and controls had five replicates. Aliquots (1 mL) were taken every 2 weeks from treatments and controls. Ethanol (1 mL) was added to inactivate samples. Samples were kept at −20°C until analysis for metalaxyl residues by liquid chromatography-tandem mass spectrometry (LC-MS/MS) and total protein content.

### Sample preparation and metalaxyl/protein quantification

The samples were centrifuged (Eppendorf, model 5415 D, USA) at 16.1 RCF for 15 min and the supernatant was filtered through a syringe filter (0.45 µm, 4 mm diameter, Millipore, USA). Quantification of analytes was made by LC-MS/MS (Sciex API 2000 triple-quadrupole MS system, Perkin-Elmer, Shelton, CT). The pellet was used for measurement of total protein content. The resulting pellet was lysed according to the protocol proposed by Massoud et al. [26]. Protein content was determined following the Microtiter Plate Protocols from BioRad [27].

### Characterization of bacterial strain by gen *ribosomal 16S* DNAr

The phenotypic characterization of the bacteria consisted of a Gram stain [28] and for the genotypic characterization we used a Wizard Genomic DNA purification kit to obtain bacterial DNA. The ribosomal gen 16S DNAr was amplified with the initiators 27f and 1492r [29] using the enzyme GoTaq DNA polymerase Promega. The polymerase chain reaction (PCR) was conducted with a thermocycler Mastercycler gradient, consisting of an initial cycle of denaturation (94°C, 5 min), 35 cycles of amplification (94°C, 0.3 min; 53°C, 0.3 min: 72°C, 1.3 min) and finally one cycle of elongation (72°C, 7 min). The fragment of the ribosomal gen 16S amplified (ca. 1.5 kb) was digested with 5 U of the restriction enzymes *Alu*I, *Hae*III y *Hha*I. The restriction profile was determined and compared to different bacterial strains with electrophoresis using 3% agarose gels. The amplified fragment of the 16S DNAr gen was cloned in the vector PCR2.1, using a commercial TA cloning kit. The sequence of the 16S DNAr gen was obtained using an Applied Biosystems sequencer (Model 3730), using the universal initiators M13 present in the vector. The sequence was compared with the NCBI data base (National Center for Biotechnology Information from USA). A phylogenetic analysis was performed, where the tree topology was inferred with the UPGMA method (Unweighted Pair Group Method with Arithmetic Mean) using the MEGA version 4 program.

### Inoculation of potato seedlings

Potato seedlings (21 day-old) were inoculated with a bacterial suspension (4×10^9^±7.549 UFC mL^−1^) following the protocol of Zuno et al. [24], Sahay and Varma [30], Barka et al. [31], Martinez et al. [32], Dini et al. [33], Anderote et al. [34] and Ma et al. [35]. The seedling containers were sealed and placed in photoperiod as previously described growth chamber. The containers were monitored daily to verify the bacterial-root association, through the formation of a biofilm surrounding the roots.

### Quantitation of endophytic bacteria in potato seedlings

The protocol of Rosenblueth and Martinez-Romero [20], Reinhold-hurek and Hurek [21] Zuno et al. [24], Dini et al. [33], Anderote et al. [34] and Ma et al. [35] was followed to determine the number of endophytic bacteria in potato seedlings (n = 5). A serial dilution plating technique was followed (until decimal dilution 1×10^5^) and a pouring plate technique was used. Potato dextrose agar medium was added (25 mL) to each petri dish and the dishes were incubated at 30°C for 24 h, after which a colony count was conducted.

### Transfer potato seedlings to Murashige & Skoog media containing metalaxyl

Under aseptic conditions, the inoculated potato seedlings were removed from the containers and the Murashige & Skoog media attached to the roots was rinsed off with sterile distilled water. Excess water was removed and the individual seedlings were deposited in sterile glass test tubes containing 5 mL of liquid Murashige and Skoog media containing metalaxyl (50 mg kg^−1^ of media). Bacteria-free potato seedlings were used as controls and were treated in the same way as inoculated seedlings. The potato seedlings were exposed to metalaxyl for 30 days. To check for potential photodegradation of metalaxyl during the photoperiod, tubes containing 5 mL of Murashige and Skoog media with metalaxyl (50 mg kg^−1^ of media) were included as controls. After 30 days, the potato seedlings were transferred to tubes containing 10 mL of Murashige & Skoog media (metalaxyl free) and incubated for another 60 days. During this time, growth parameters were recorded (fresh weight, length increase and vigor). For treatments and controls, 10 replicates (consisting of one potato seedling) were included. Treatment groups consisted of seedlings that were inoculated with bacteria (PB) and inoculated with bacteria plus metalaxyl (PMB). The control group consisted of seedlings without any treatment (P) and a treatment only with metalaxyl (PM).

### Sample preparation and extraction of metalaxyl and its acid metabolite

Metalaxyl and its acid metabolite were extracted from potato seedlings (n = 3) by adding 10 mL of acetonitrile to 1 g of sample. The samples were homogenized (UltraTurrax model T25, Janke & Kunkel IKA-Labortechnik, Germany) for 3 min at 13,500 rpm. The extract was cleaned using a C_18_ cartridge (6 mL, Supelclean, Sigma Aldrich, Germany). The extract was collected and concentrated to 0.5 mL with nitrogen at 30°C. Each extract was then filtered (0.45 µm, 4 mm diameter, Millipore, USA), placed into a vial and stored at −20°C until analyzed by LC-MS/MS.

To extract metalaxyl from Murashige and Skoog media, aliquots of media (1 mL) were centrifuged in Eppendorf tubes at 16.1 RCF for 20 min, filtered (0.45 µm, 4 mm diameter, Millipore, USA) and stored at −20°C until analyzed by LC-MS/MS.

### Instrumental Conditions

Metalaxyl and its acid metabolite were analyzed using an AB Sciex API 2000 triple-quadrupole-tandem mass spectrometer (AB Sciex Instruments, Foster City, CA, USA) with Atmospheric Pressure Chemical Ionization (APCI). The instrument was controlled using “Analyst” software (version 1.4.2; Applied Biosystems, Foster City, CA). The high performance liquid chromatography (HPLC) equipment consisted of a Hewlett-Packard Model 1100 series with a quaternary pump system, auto sampler and in-line degasser. The analytes were separated using a C_18_ VARIAN Pursuit column (100×3.0 mm, i.d., 3 µm particle size, Santa Clara, CA) fitted to a C_18_ pre-column (4×3.0 mm i.d., Phenomenex, Torrance, CA). The injection volume was 10 µL and the HPLC was operated at a flow rate of 600 µL min^−1^. A gradient program was used, consisting of 60% water/40% acetonitrile/0.1% acetic acid for 0.50 min, followed by 10% water/90% acetonitrile/0.1% acetic acid for 4.0 min, and 60% water/40% acetonitrile/0.1% acetic acid for 4.0 min. The total run time per sample was 8.0 min. The retention times were 2.56 min for metalaxyl and 1.6 min for the acid metabolite. The system configuration was as follows: temperature 425°C, dwell time 300 ms and nebulizing current 3 kV. The curtain gas, gas 1, gas 2 were set at 35, 40 and 15, respectively.

The declustering potential (DP), focusing potential (FP), entrance potential (EP), collision energy (CE), collision cell exit potential (CXP), and collision cell entrance potential (CEP) for each compound are listed in Table 1. Quantitation of metalaxyl and its acid metabolite was done using external standards by comparison to a ten and eight point calibration curve, respectively. The area of the analyte response was plotted against the amount of analyte injected. A linear regression was used to generate calibration curves over a range of concentrations (0.20–100 mg kg^−1^, metalaxyl, and 0.2–20 mg kg^−1^, acid metabolite. These ranges spanned the concentration range found in potato seedlings and in all cases r^2^>0.999. MRM parameters were developed by infusing 100 µg mL^−1^ solutions of each compound into the MS with mobile phase at flow rate of 25 µg mL^−1^. The most abundant transition ion with the lowest accompanying background was selected for each analyte (280.1→220.1 for metalaxyl and 266.1→206.1 for the acid metabolite). Metalaxyl and its acid metabolite had LODs of 0.2 mg kg^−1^ and 0.04 mg kg^−1^, respectively. Metalaxyl and its acid metabolite had LOQs of 0.4 mg kg^−1^ and 0.3 mg kg^−1^, respectively. These results were generated with intra-assay (same day) coefficients of variation less than 5%. In the presence of sample matrix, the metalaxyl acid metabolite showed a gain of 50% in signal due to ion enhancement. To overcome this matrix effect, accurate quantitation of this analyte was achieved by the preparation of a standard curve in the matrix.

**Table 1:** Analytical LC-MS/MS conditions.

Analyte	Ion	Transition	DP^a^	FP^b^	EP^c^	CE^d^	CXP^e^	CEP^f^
						(V)		
Metalaxyl	Quantification	280.1→220.1	16	360	12	19.00	10.00	30.00
Metalaxyl	Confirming	280.1→192.1	16	360	12	23.00	8.00	30.00
Acid metabolite	Quantification	266.1→206.1	21	360	12	21.00	4.00	30.00

a Declustering potential.

b Focusing potential.

c Entrance potential.

d Collision energy.

e Collision cell exit potential.

f Collision cell entrance potential.

### Metabolite Profiling by GC–TOF–MS

The extraction and analysis of potato seedlings for metabolomic profiling were carried out at the Metabolomics Core, Genome Center of University of California, Davis (Davis, CA, USA). Seedlings were harvested after 30 days of exposure to metalaxyl. Each group consisted of 10 seedlings. Sample preparation and extraction followed the protocol reported by Fiehn et al. [36]. Briefly, seedlings (∼30 mg) for each treatment were harvested and transferred to a 2 mL Eppendorf tube, immediately frozen in liquid nitrogen and crushed in a Restch∼mill (Hann, Germany). A 1 mL aliquot of pre-chilled extraction solvent mixture (acetonitrile∶isopropanol∶water 3∶3∶2) at −20°C was added, and the pH was adjusted between 5 and 6. The samples were vortexed for about 10 s at room temperature and then shaken for 4–6 min at 4°C using an orbital mixing chilling/heating plate (Thermomixer R., Cole-Parmer, USA). The tubes were centrifuged for 2 min at 14,000 g. The supernatant was decanted into a new Eppendorf tube and the extracts were evaporated to complete dryness overnight at room temperature (26°C) using a Labconco Centrivap cold trap concentrator (Labconco, Corporation, Kansas City, MO). An aliquot of 500 µL of acetonitrile∶water (50% v/v) was added and the extracts were vortexed. The extracts were centrifuged at 14,000 g, 4°C for 3 min. The eluent was transferred to a new Eppendorf tube and evaporated to dryness overnight at room temperature. (26°C) using a Labconco Centrivap cold trap concentrator. To the dried samples 20 µL of 40 mg/mL methoxylamine hydrochloride in pyridine was added, and tubes with the samples were agitated at 30°C for 30 min. Subsequently, 180 µL of trimethylsilylating agent *N*-methyl-*N*-trimethylsilyltrifluoroacetamide (MSTFA) was added, and samples were agitated at 37°C for 30 min. GC–TOF–MS analysis was performed using an Agilent 6890 N gas chromatograph (Palo Alto, CA, USA) interfaced to a time-of-flight (TOF) Pegasus III mass spectrometer (Leco, St. Joseph, MI). Automated injections were performed with a programmable robotic Gerstel MPS2 multipurpose sampler (Mülheim an der Ruhr, Germany). The GC was fitted with both an Agilent injector and a Gerstel temperature-programmed injector, cooled injection system (model CIS 4), with a Peltier cooling source. An automated liner exchange (ALEX) designed by Gerstel was used to eliminate cross-contamination from sample matrix occurring between sample runs. Multiple baffled liners for the GC inlet were deactivated with 1- µL injections of MSTFA. The Agilent injector temperature was held constant at 250°C while the Gerstel injector was programmed (initial temperature 50°C, hold 0.1 min, and increased at a rate of 10°C/s to a final temperature of 330°C, hold time 10 min). Injections of 1 µL were made in split (1∶5) mode (purge time 120 s, purge flow 40 ml/min). Chromatography was performed on an Rtx-5Sil MS column (30 m×0.25 mm i.d., 0.25 µm film thickness) with an Integra-Guard column (Restek, Bellefonte, PA). Helium carrier gas was used at a constant flow of 1 mL/min.

The GC oven temperature program was 50°C initial temperature with 1 min hold time and ramping at 20°C/min to a final temperature of 330°C with 5 min hold time. Both the transfer line and source temperatures were 250°C. The Pegasus III TOF (Leco, St. Joseph, MI) mass spectrometer ion source operated at −70 kV filament voltage with ion source. After a solvent delay of 350 s, mass spectra were acquired at 20 scans per second with a mass range of 50 to 500 *m*/*z*. Resulting GC-TOF-MS data were processed following the methods outlined by Fiehn et al. [36]. In brief, initial GC-TOF-MS peak detection and mass spectrum deconvolution were performed with ChromaTOF software version 2.25 (Leco). A reference chromatogram was defined that had a maximum of detected peaks over a signal/noise threshold of 20 and used for automated peak identification based on mass spectral comparison to standard and in-house customized mass spectral libraries. Mass spectra were searched against custom spectrum libraries (e.g. the Fiehn library of 713 unique metabolites) and identified based on retention index and spectrum similarity match. All known artifactual peaks caused by column bleeding or phthalates and polysiloxanes derived from *N*-methyl-*N*-trifluoroacetamide (MSTFA) hydrolysis were manually identified and removed. Resulting data for each sample were normalized using the total summed metabolite concentration and then logarithmically transformed (base = 10). For each metabolite, transformed values greater than six standard deviations from mean across sample groups were set to missing data.

### Statistical Analysis

Processing of the raw data yielded 137 identified metabolites from potato seedling samples. Statistical analysis was applied to GCMS data from P, PB, PM and PMB sample groups. Statistical analysis was performed by the submission of previously normalized data to web based service for metabolomic data analysis: MetaboAnalyst (http://www.metaboanalyst.ca/MetaboAnalyst/faces/Home.jsp). Additional statistical analysis was performed with the use of MarkerView™ software versión 1.2 (AB Sciex, Foster City, CA, USA) and SAS Software version 9.0 (SAS Institute Inc., Cary, NC, USA). The primary objective of the statistical analysis was to identity metabolites whose concentrations differentiate significantly between control (untreated) plants and infected/treated plants. To identify metabolites as metabolic markers, we wanted to identify metabolites which were capable of sample groups' discrimination, and further to identify sets of relevant metabolites that act synergistically within functionally defined pathways. Specifically, to identify metabolites whose expression was associated with the plant treatment, we performed differential analysis based on a general linear model. Analysis of variance (ANOVA) was used to assess groups' effects for each metabolite. Unsupervised principal component analysis (PCA) was applied that best explain the variance in a data set without referring to group labels. Supervised discriminant analysis PLS-DA (MetaboAnalyst) and PCA-DA (MarkerView™ 1.2) which use multivariate regression techniques to extract via linear combination of original variables the information that can predict the group membership was further applied to visualize samples clustering and identify the most important features.

### Pathway Analysis

Pathway analysis was performed utilizing MetPA: A web-based metabolomics tool for pathway analysis & visualization (http://metpa.metabolomics.ca/MetPA). MetPA (Metabolomic Pathway Analysis) is a web-based tool that combines result from pathway enrichment analysis with the pathway topology analysis which allowed identifying the most relevant pathways involved in the conditions under currently reported study. Data for identified metabolites detected in all samples was submitted into MetPA with annotation based on common chemical names. Verification of accepted metabolites was conducted manually using HMDB, KEGG, and PubChem DBs. Arabidopsis thaliana (thale cress) pathway library was used for pathway analysis. List of the most impacted pathways was generated in accordance with previously described approaches [37].

## Results and Discussion

**Figure 1:**
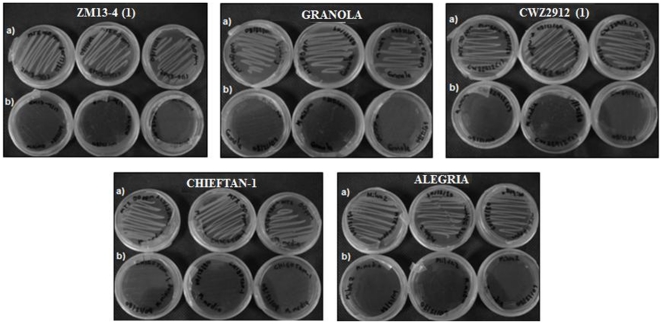
Growth of rhizospheric bacteria isolated from potato plants on minimal liquid media. (**A**) Minimal liquid media with metalaxyl (50 mg kg^−1^ of media). (**B**) Minimal liquid media.

**Figure 2:**
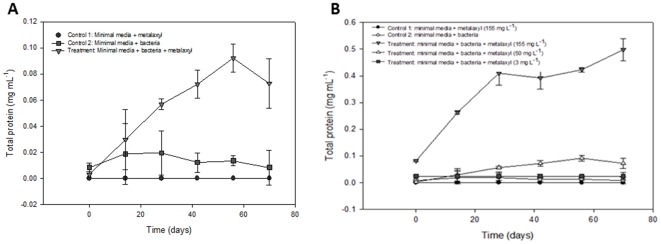
Effect of metalaxyl on microbial growth. (**A**) Effect of metalaxyl (50 mg kg^−1^) on microbial growth in minimal liquid media. (**B**) Effect of metalaxyl on microbial growth in minimal liquid media. Each point data represents an average of 5 repetitions (± standard deviation).

**Figure 3:**
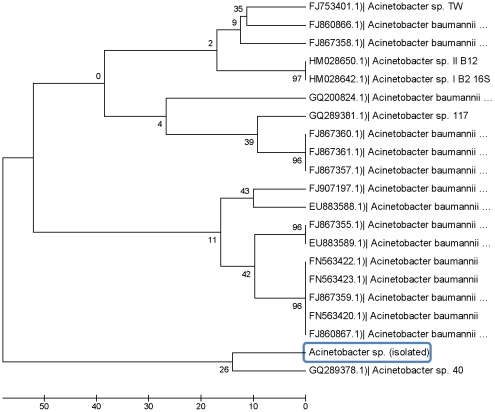
Phylogenetic tree based on the 16S DNAr gen analysis of the *Acinetobacter* genera. The isolated bacterium is enclosed in blue. The bar represents five substitutions of nucleotides for 1000 nucleotides. The robustness of the tree was determined using 1000 replicates of bootstrap and the access number to the GenBank of every strain is shown.

Samples of five varieties of potato plants (ZM#13-4, CW2912, Alegria, Chieftan and Granola) were collected from potato fields with a history use of metalaxyl application. Five isolates were obtained and their ability to grow in the presence of metalaxyl was assessed by culturing the strains on a minimal media containing metalaxyl. Controls were grown in media without metalaxyl. Each strain and corresponding control included three replicates. After incubating for 24 h at 37°C, strains only grew on media that contained metalaxyl (Figure 1). The bacterial strain isolated from the Chieftan potato plant variety was evaluated for metalaxyl degradation in minimal liquid media. This strain showed a 5-fold increase in total protein content compared to growth in the absence of metalaxyl (Figure 2). The increase in protein content for the strain isolated from ZM#13-4, CW2912, Alegria, and Granola potato varieties was 3 times less than strains isolated from the Chieftan variety (data not shown). The gram stain showed that the bacteria isolated from the Chieftan potato plant variety was a Gram negative coco-bacillus. The genotypic characterization indicated that the bacterial strain had a 98% resemblance to the strain *Acinetobacter* sp (FJ753401.1). A phylogenetic analysis showed that the closest strain to the one obtained from the potato fields also had a 98% similarity to *Acinetobacter* sp 40 (GQ289378) (Figure 3). Although an increase in total protein content was observed for the strain isolated from the Chieftan variety, concentration of metalaxyl in the media did not decrease over time (data not shown). It's important to note that the bacterial strain was exposed to three different metalaxyl concentrations in minimal media (155, 50 and 3 mg L^−1^, Figure 2B). It was observed that as the metalaxyl concentration increased; microbial growth was favored, reflecting the increase in total protein content, from 0.082±0.003 to 0.498±0.041 mg mL^−1^ at the metalaxyl concentration of 155 mg L^−1^. This behavior was observed by Pooja et al. [38], Liu et al. [39], Xie et al. [40], Hongsawat and Vangnai [41] and Chanika et al. [42], when they performed similar experiments with species of *Acinetobacter* to degrade different pesticides. There are studies that report the use of *Acinetobacter* to degrade pesticides such as atrazine [38], methyl parathion [39], malathion [40], and chloroanilines [41]. However, no studies mention the use of *Acinetobacter* sp in metalaxyl degradation, although this is a common soil microorganism [38]. Bailey and Coffey [23] studied different bacterial strains for metalaxyl degradation and showed that eight strains of fungi and six strains of bacteria degraded metalaxyl in liquid medium.

### Inoculation of potato seedlings with *Acinetobacter* sp

**Figure 4:**
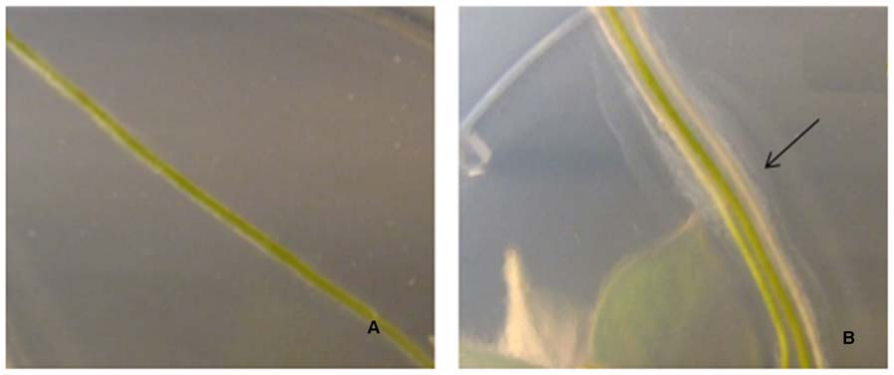
Potato seedling roots. (**A**) Seedlings control. (**B**) Seedling inoculated with *Acinetobacter* sp. Arrow points to biofilm formation of *Acinetobacter sp* surrounding potato roots 3 days after inoculation.

**Figure 5:**
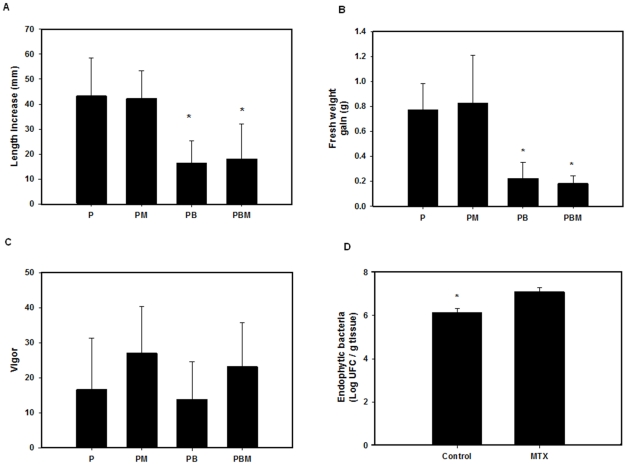
Effects of metalaxyl on growth of potato seedlings after a 30 day exposure period. (**A**) Effect of metalaxyl on length increase. (**B**) Effect of metalaxyl on fresh weight gain. (**C**) Effect of metalaxyl on vigor. (**D**) Effect of metalaxyl on the number of endophytic bacteria. Bars represent standard deviation of the mean (n = 10). *Significantly different from controls (p≤0.05).

*Acinetobacter* sp formed a bacterial biofilm around the potato seedling roots 3 days after inoculation (Figure 4). Several studies have determined the period of time that different bacteria with endophytic potential may need in order to associate the plant roots [43], *Pseudomona fluorescens* SBW25 associates with wheat roots in a period of 6 to 9 days. Garcia et al. [44], determined that *Azospirillum* spp and *Azotobacter beijerincki* colonized wheat Pavon variety after 3 days. Prieto and Mercado-Blanco [45], found that *Pseudomonas fluorescens* (PICFZ) colonized olive roots in 9 days. Zuno et al. [24] found that *Pseudomonas* sp takes 7 days to associate with potato Seedling Alpha variety and Rainey [46] reported that root association of different *Pseudomonas* strains took 14 days after inoculation. According to these studies, the time that a microorganism needs to associate with plant roots depends on many factors; one of the most important is the affinity that a microorganism has for the exudates liberated by the plant [47], [48]. The population density of *Acinetobacter* sp in the interior of potato seedlings was observed after the root-bacteria association (three days) and 3.93±0.865 log CFU g^−1^ of vegetable tissue was observed. After 30 days of incubation of potato seedlings in Murashige and Skoog with and without metalaxyl, the population density of cells increased to an average concentration of 7.10±0.2 log CFU g^−1^ of vegetable tissue and 6.13±0.2 log CFU g^−1^ of vegetable tissue, respectively (Figure 5D).

Statistical analysis indicated that the density of cells of *Acinetobacter* sp in the treated potato seedlings with metalaxyl was 14% higher and significantly different than the control group (p≤0.05). This value remained constant for several weeks until the conclusion of the experiment. The population densities of *Acinetobacter* sp in potato seedlings were similar to those reported by other investigators. Sturz and Nowak [49], found a population density of 3 to 8 log CFU g^−1^ of vegetable tissue. When working with strains of *Pseudomonas*, Germaine et al. [50] reported a bacterial density in poplar plants of 4 to 5 log CFU g^−1^ of vegetable tissue. Adachi et al. [51] found a population of 3 to 5 log CFU g^−1^ of vegetable tissue in sweet potato. Garbeva et al. [52] reported a concentration of endophytic bacteria in potato seedlings of 4 to 6 log CFU g^−1^ of vegetable tissue. Elvira-Recuenco and van Vuurde [53] found values of 4 to 8 log CFU g^−1^ of vegetable tissue in pear cultivars and Zuno et al. [24] reported a population of 5.28 log CFU g^−1^ for *Pseudomonas* sp in potato seedlings Alpha variety.

The results obtained in the present study indicate that *Acinetobacter* sp was capable of establishing as a putative endophyte in potato seedlings. However further confirmation that *Acinetobacter* sp is a true endophyte of potato seedlings is needed using techniques as immunological detection of bacteria, fluorescence tags, confocal laser scanning microscopy or specific oligonucleotide probes [54], [55], [56].

### Response of potato seedlings to metalaxyl and *Acinetobacter* sp

**Figure 6:**
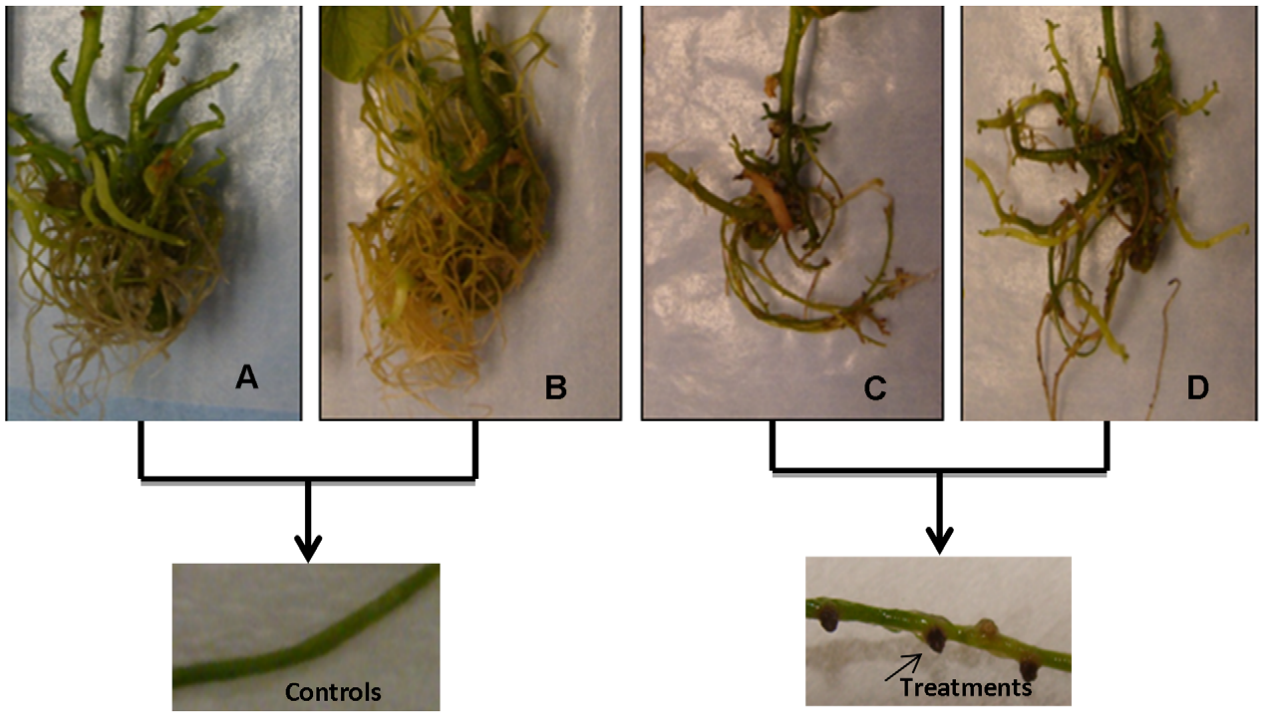
Effects of *Acinetobacter* sp on root formation in potato seedlings 30 days after inoculation. (**A**) Control potato seedling. (**B**) Potato seedlings exposed to metalaxyl (50 mg kg^−1^). (**C**) Potato seedlings inoculated with *Acinetobacter* sp. (**D**) Potato seedlings inoculated with *Acinetobacter* sp and exposed to metalaxyl (50 mg kg^−1^). Arrows point to the effects of *Acinetobacter* sp on the potato roots.

Our results suggest that metalaxyl did not affect the growth of potato seedlings during 30 days of exposure (Figure 5A, B and C). The potato seedling length increase, fresh weight gain and vigor were similar in both treated seedlings and controls. Although metalaxyl did not affect the growth of potato seedlings, it did result in a 14% increase in the growth of *Acinetobacter* sp inside the potato seedlings compared to controls (Figure 5D). *Acinetobacter* sp, in turn, strongly affected potato seedling growth, as confirmed by a 40% reduction in length and fresh weight (27%). *Acinetobacter* sp also affected root formation (Figure 6). Potato seedlings inoculated with bacteria showed a decrease in root formation compared to the controls. This effect on growth could be due to change in phytohormone production as reported earlier, however further elaborate studies are necessary to corroborate these findings. [57], [58], [59], [60], [61]

Small cay formed on the roots of the potato seedlings inoculated with *Acinetobacter* sp, giving the appearance of nodules. Potato seedlings that were not inoculated were not affected in such a manner (Figure 6). One of the effects observed in the inoculated seedlings was an early tuberization; inoculated potato seedling produced at least 1±1 tuber at 75 days compare to controls (no tuber). Significant statistical differences were observed between inoculated seedlings and controls when data was analyzed by the Kruskal Wallis test (p<0.0001). The production of tubers in inoculated seedlings was initiated one month before the normal cycle of potato production (three months). It is necessary to conduct studies to determine the effect of *Acinetobacter* sp on some metabolic pathways in potato seedlings which may have influence on tuber production [62], [63], [64], [65]. The results obtained in the present work are similar to those reported by Frommel et al. [66], Nowak et al. [67] and Sturz [68].

**Figure 7:**
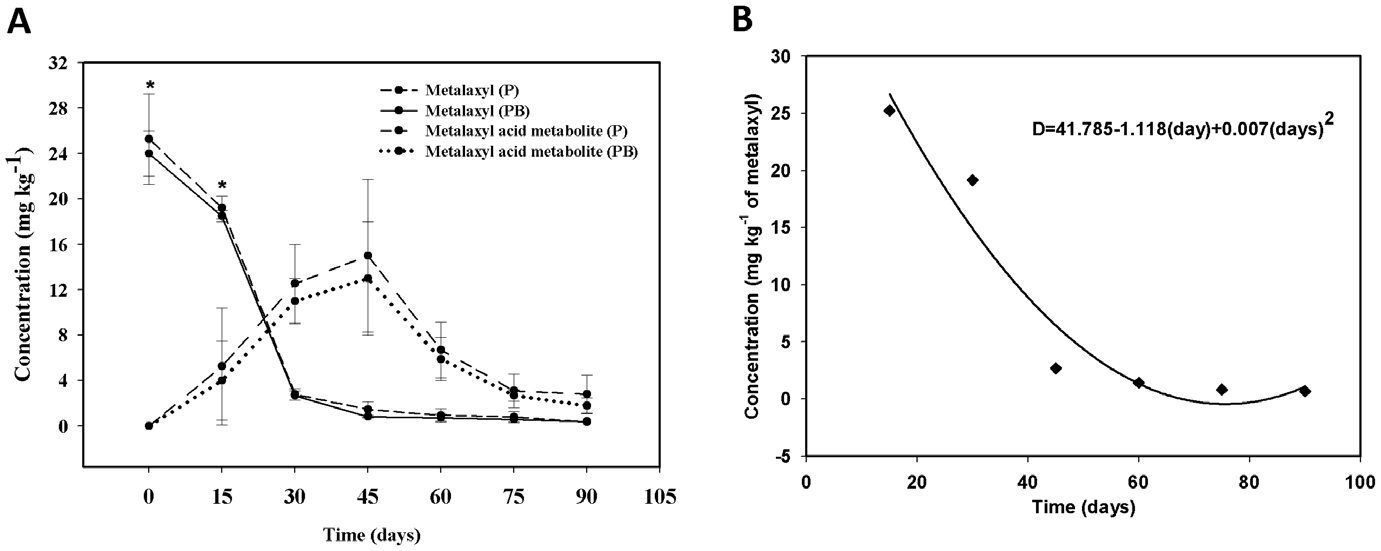
Degradation of metalaxyl in potato seedlings. (**A**) Concentration of metalaxyl and formation of acid metabolite in potato seedlings over time (P = Potato seedlings, PB = Potato seedlings inoculated with *Acinetobacter* sp). (**B**) Quadratic model of metalaxyl degradation in potato seedlings over time, the standard deviation was 3.472 and F value was 23.098. * *P*<0.05. Each point represents an average of 3 repetitions standard deviation.

LC-MS/MS was used to analyze metalaxyl and the metalaxyl acid metabolite. The SPE percentage recovery at 50 mg kg^−1^ was 95±9% (n = 5) for metalaxyl and 92±9% (n = 5, 50 mg kg^−1^) for acid metabolite. The results obtained by LC-MS/MS indicated that *Acinetobacter* sp did not contribute to metalaxyl degradation in potato seedlings (Figure 7A). According to these results the degradation behavior of metalaxyl in potato seedlings follows a quadratic model with an r^2^ of 0.93 (Figure 7B). As shown in Figure 7A, the maximum concentration of metalaxyl absorbed by the seedlings was 22±4 mg kg^−1^ after 15 days of exposure. This was reduced by 97% after 90 days (0.66±0.29 mg kg^−1^ of metalaxyl). Mehta et al. [69], found that 9 mg kg^−1^ in mustard plants completely dissipated in 60 days. Zadra et al. [15], measured 2.7 mg kg^−1^ of metalaxyl in sunflower plants 38 days exposure of metalaxyl. This measurement decreased by 67% to 0.9 mg kg^−1^ after 85 days. Badaway et al. [70] reported a concentration of 19 mg kg^−1^ of metalaxyl which was reduced by 82% to 0.49 mg kg^−1^ after 7 days. Overall metalaxyl degrades anywhere from 67% to 100% over a period of 7 to 60 days.

The highest concentration of acid metabolite found in vegetable tissue was 15±7 mg kg^−1^ after 45 days of exposure to metalaxyl. This concentration was reduced by 81% to 2.8±1.7 mg kg^−1^ of acid metabolite after 90 days (Figure 7A). In studies performed with ^14^C-metalaxyl did not detect the presence of acid metabolite in potato foliage, but there were found 2.8 mg kg^−1^ of acid metabolite in tubers [71]. Stingelin (cited by Hamilton [71]) reported a concentration of 0.25 mg kg^−1^ of acid metabolite in lettuce plants 14 days after exposure decreasing 8% in 21 days to 0.02 mg kg^−1^. Zadra et al. [15] observed the formation of acid metabolite in sunflower plants 21 days after treatment with metalaxyl, reaching a concentration of 1.3 mg kg^−1^ after 85 days of exposure. Acid metabolite shows a clear tendency to degrade over time inside the potato seedlings, which may suggest that it's only one of several metabolites reported to be involved in various metalaxyl degradation routes in plants [6], [7].

### Statistical Analysis of the Metabolite Profiling Data

**Figure 8:**
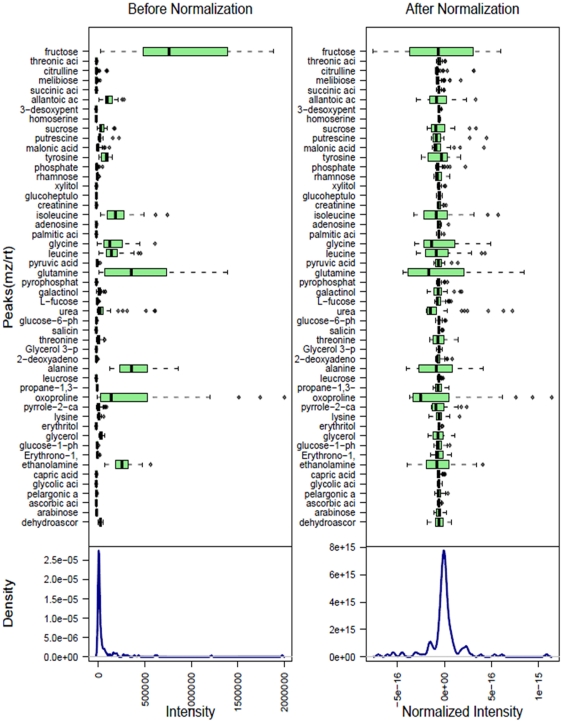
Data normalization in MetaboAnalyst. Box plots and kernel density plots before and after normalization. The boxplots show at most 50 features due to space limits. The density plots are based on all samples. Selected methods: Row-wise normalization: Normalization to constant sum, Column-wise normalization: Pareto Scaling.

**Figure 9:**
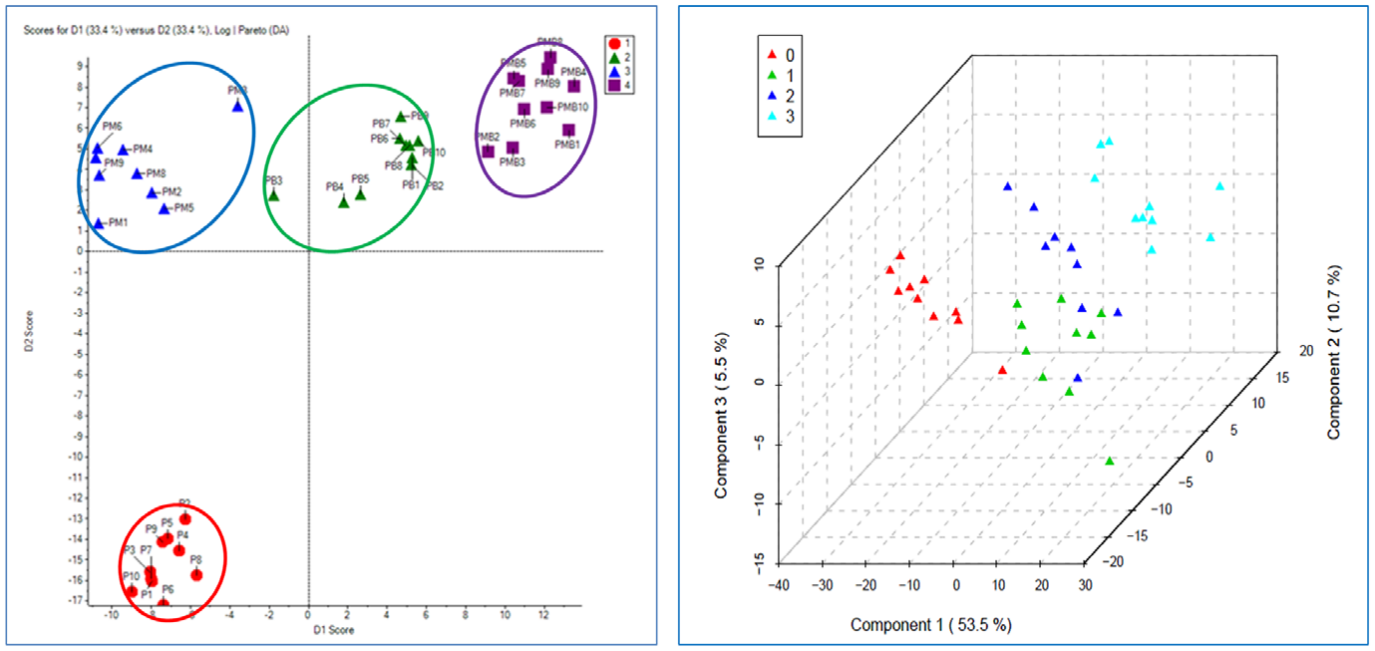
PCA-DA (MarkerView) and PLS-DA (MetaboAnalyst) score plots of discriminant analysis. Group labels: P = 1 and 0, PB = 2 and 1, PM = 3 and 2, PMB = 4 and 3.

**Figure 10:**
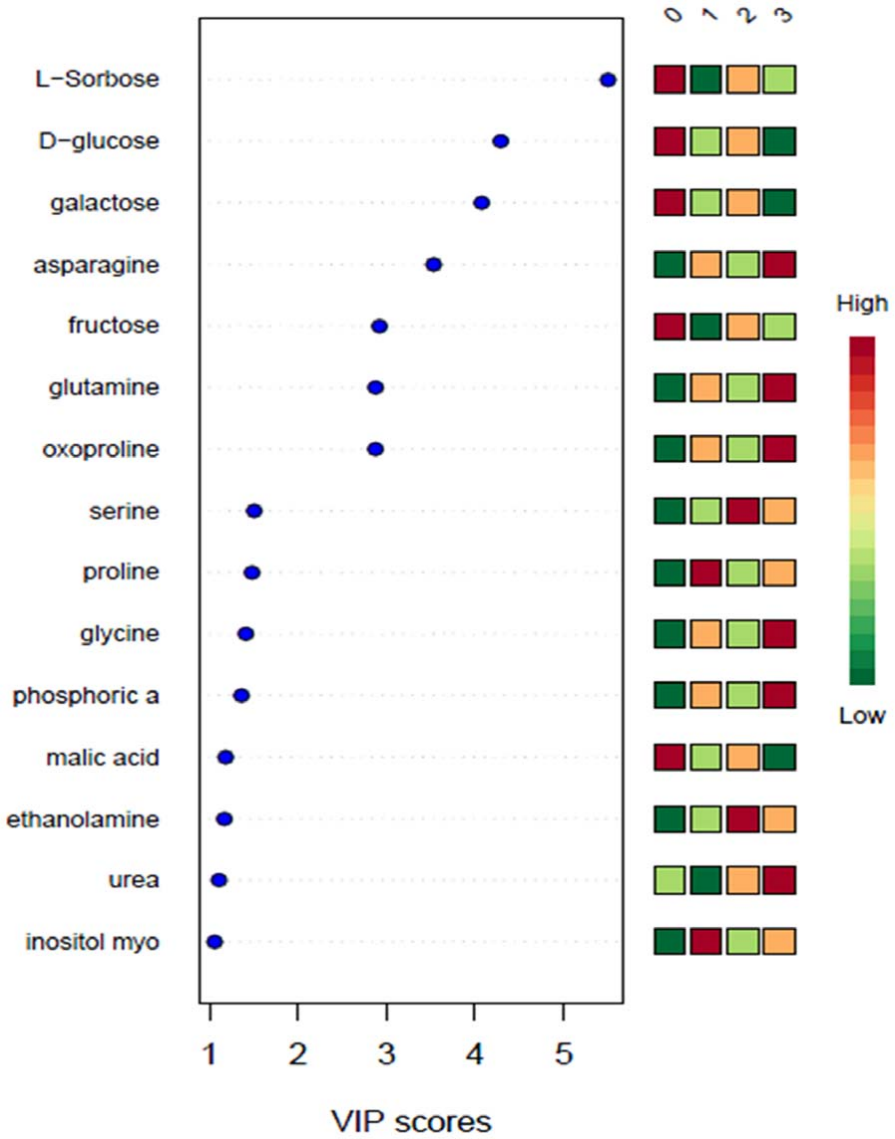
Important features (candidates to metabolic markers) identified by PLS-DA (MetaboAnalyst). The colored boxes on the right indicate the relative concentrations of the corresponding metabolite in each group under current study. Group labels: P = 0, PB = 1, PM = 2, PMB = 3.

**Figure 11:**
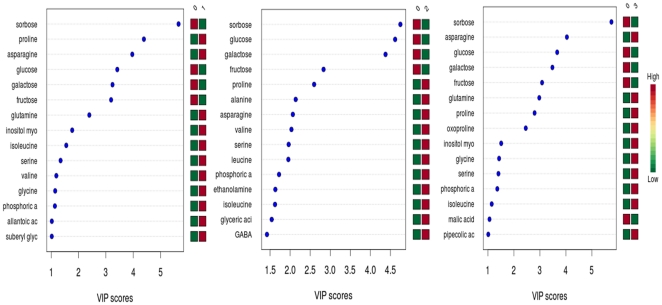
Important features (candidates to metabolic markers) identified by PLS-DA (MetaboAnalyst) during side by side analysis. The colored boxes on the right indicate the relative concentrations of the corresponding metabolite in each group under current study. Group labels: P = 0, PB = 1, PM = 2, PMB = 3.

Metabolite profiling is a powerful tool that has contributed to the understanding of plant physiology, including phenotypic differences, gene annotations, metabolite regulation, and characterization of stress responses. Metabolic profiling of potato tubers has been accomplished using NMR, HPLC-UV and GC-MS techniques [72], [73], [74]. In the present research one study statistical analysis was used to investigate modifications to metabolic pathways. However, metabolic profiling of potato seedlings, as well as effect of *Acinetobacter* sp and metalaxyl on metabolic profiles of potato seedlings was never carried out. Data normalization results, performed with MetaboAnalyst assistance prior statistical analysis, are shown on Figure 8. PCA (not shown), PCA-DA and PLS-DA were applied to normalized GC-MS data from P, PB, PM and PMB sample groups in order to identify possible variations in the metabolite composition between the infected and non-infected samples. Out of the 18 principal components, the first three (PC1, PC2 and PC3) were responsible for major variation (67.6%) in study groups. Score plots clearly demonstrated that all four groups of samples, P, PB, PM and PMB cluster into four very distinct groups (Figure 9). Further PCA-Discriminant Analysis resulted in three components, which contributed equally to 100% of variation. The list of 15 the most important features is shown on Figure 10 alone with the indication of relative metabolite concentrations in each group. When groups compared side by side, there are metabolites that having larger score in the particular pare (Figure 11). For instance proline and asparagine taking place of glucose in the line of important features illustrating changes characteristic for each group of samples (see Figure 11).

### Metabolic Pathway Analysis

**Figure 12:**
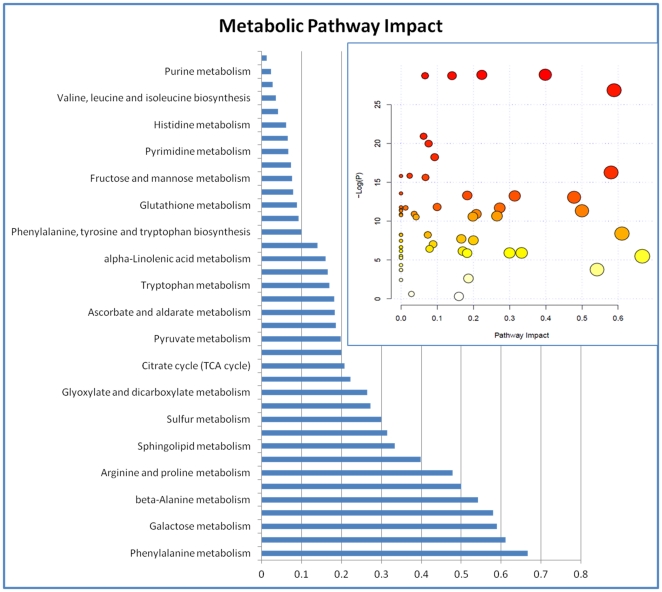
Metabolic Pathway Impact overview generated by MetPA. Unaltered pathways have score 0. The most impacted having high statistical significance score colored red.

**Figure 13:**
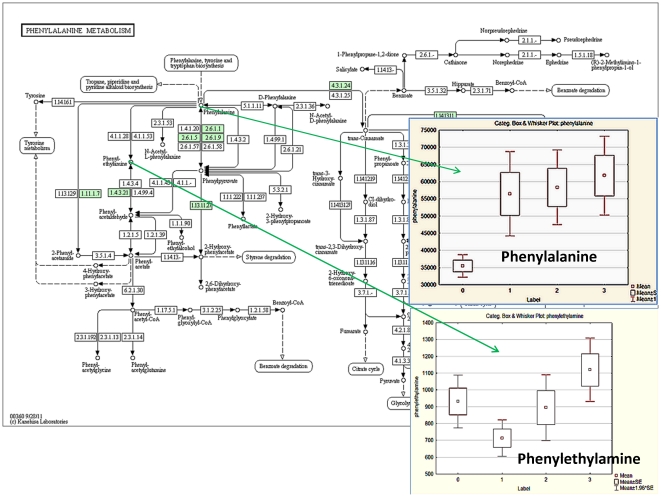
Phenylalanine metabolism chart with Box-Whisker plots for Pnenylalanine and Phenylethylamine. Group labels: P = 0, PB = 1, PM = 2, PMB = 3.

**Figure 14:**
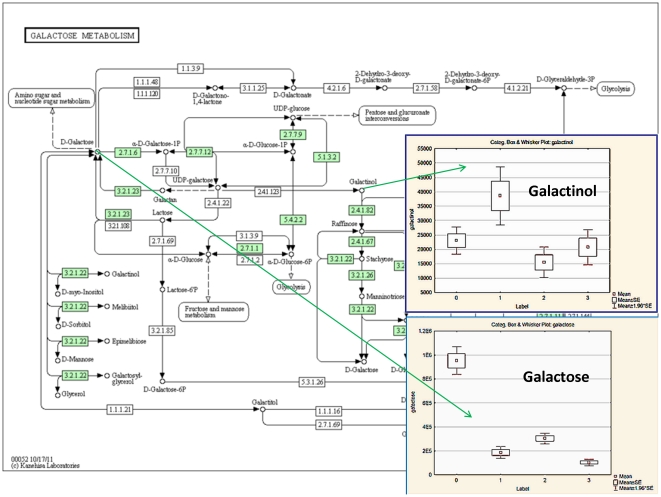
Galactose metabolism chart with Box-Whisker plots for Galactosee and Galactinol. Group labels: P = 0, PB = 1, PM = 2, PMB = 3.

Pathway analysis performed with MetPA assistance revealed a number impacted metabolic pathways. The overall pathway impact picture is illustrated with Figure 12. This data presented in greater details in Table 2 where number of hits, p values and KEGG links are depicted. The most impacted appears to be phenylalanine metabolism; glycine, serine and threonine metabolism; galactose metabolism; alanine, aspartate and glutamate metabolism, etc. However impact consequences are not straightforward and metabolic flux is involved. Changes in flux can be illustrated with Figure 13 and Figure 14 where some metabolites do not follow the pattern of key metabolite. This indicates flux redistribution upon influence of bacterial infection and influence of Metalaxyl and its metabolites. This confirms a high complexity and sensitivity of metabolic networks exploited by plant in order to survive and adjust to environmental challenges.

**Table 2:** Metabolic Pathway Impact table generated by MetPA.

Pathway Name	Total Cm pd	Hits	Raw p	Holm p	FDR	Impact	Details
Phenylalanine metabolism	8	2	0.0040895	0.052246	0.0049287	0.66667	KEGG
Glycine, serine and threonine metabolism	30	6	2.21E-04	0.0063993	4.20E-04	0.61049	KEGG
Galactose metabolism	26	11	2.15E-12	1.18E-10	2.53E-11	0.58877	KEGG
Alanine, aspartate and glutamate metabolism	22	7	8.46E-08	4.31E-06	5.54E-07	0.58046	KEGG
beta-Alanine metabolism	12	1	0.022737	0.15916	0.025312	0.54167	KEGG
Isoquinoline alkaloid biosynthesis	6	1	1.18E-05	4.47E-04	3.03E-05	0.5	KEGG
Arginine and proline metabolism	38	11	2.06E-06	9.08E-05	7.61E-06	0.4783	KEGG
Starch and sucrose metabolism	30	4	2.93E-13	1.73E-11	4.90E-12	0.3988	KEGG
Sphingolipid metabolism	13	2	0.0026698	0.042717	0.0035069	0.33333	KEGG
Inositol phosphate metabolism	24	2	1.74E-06	7.85E-05	6.86E-06	0.31414	KEGG
Sulfur metabolism	12	2	0.0026747	0.042717	0.0035069	0.3	KEGG
Tyrosine metabolism	18	3	8.12E-06	3.33E-04	2.41E-05	0.27273	KEGG
Glyoxylate and dicarboxylate metabolism	17	4	2.34E-05	7.47E-04	4.92E-05	0.2653	KEGG
Amino sugar and nucleotide sugar metabolism	41	5	2.99E-13	1.73E-11	4.90E-12	0.22331	KEGG
Citrate cycle (TCA cycle)	20	5	1.79E-05	6.45E-04	4.24E-05	0.20803	KEGG
Pantothenate and CoA biosynthesis	14	3	5.29E-04	0.012686	8.66E-04	0.2	KEGG
Pyruvate metabolism	21	3	2.51E-05	7.78E-04	5.11E-05	0.19856	KEGG
Glycerophospholipid metabolism	25	3	0.071676	0.2867	0.075516	0.18636	KEGG
Ascorbate and aldarate metabolism	15	3	1.64E-06	7.56E-05	6.86E-06	0.1831	KEGG
Cysteine and methionine metabolism	34	6	0.0027601	0.042717	0.0035401	0.1827	KEGG
Tryptophan metabolism	27	1	0.0021229	0.038212	0.0029128	0.17059	KEGG
Methane metabolism	11	2	4.40E-04	0.01099	7.41E-04	0.16667	KEGG
Alpha-Linolenic acid metabolism	23	1	0.72217	1	0.72217	0.16	KEGG
Glycolysis or Gluconeogenesis	25	4	3.31E-13	1.89E-11	4.90E-12	0.14103	KEGG
Phenylalanine, tyrosine and tryptophan biosynthesis	21	4	7.25E-06	3.12E-04	2.41E-05	0.09982	KEGG
Aminoacyl-tRNA-biosynthesis	67	16	1.19E-08	6.18E-07	8.77E-08	0.09302	KEGG
Glutathione metabolism	26	5	8.77E-04	0.019303	0.0013623	0.08864	KEGG
Glycerolipid metabolism	13	3	0.0015921	0.030249	0.002291	0.07895	KEGG
Fructose and mannose metabolism	16	1	2.08E-09	1.10E-07	1.75E-08	0.07627	KEGG
Lysine biosynthesis	10	2	2.66E-04	0.0071798	4.62E-04	0.07407	KEGG
Pyrimidine metabolism	38	4	1.62E-07	7.76E-06	7.95E-07	0.06775	KEGG
Pentose phosphate pathway	18	3	3.32E-13	1.89E-11	4.90E-12	0.06649	KEGG
Histidine metabolism	16	1	8.05E-10	4.35E-08	7.92E-09	0.0625	KEGG
Carbon fixation in photosynthetic organism	21	3	2.65E-05	7.96E-04	5.22E-05	0.04181	KEGG
Valine, leucine and isoleucine biosynthesis	26	5	1.80E-05	6.45E-04	4.24E-05	0.03645	KEGG
Steroid biosynthesis	36	1	0.53225	1	0.54143	0.02881	KEGG
Purine metabolism	61	6	1.31E-07	6.57E-06	7.23E-07	0.02398	KEGG
Selenoamino acid metabolism	19	1	8.17E-06	3.33E-04	2.41E-05	0.01272	KEGG

Unaltered pathways have score 0.

In conclusion, the effect of *Acinetobacter* sp on metalaxyl degradation in potato seedlings as well the effect of bacteria and metalaxyl on growth of potato seedlings was investigated using GC–TOF–MS and LC-MS/MS techniques. Results from our study suggest that metalaxyl alone did not affect the growth of potato seedlings. However, *Acinetobacter* sp strongly affected the growth and root formation of inoculated seedlings. LC-MS/MS analysis of metalaxyl residues in potato seedlings suggests that *Acinetobacter* sp did not degrade metalaxyl in potato seedlings. Based on a review of the literature, we report for the first time that metabolic profiling study followed by statistical and metabolic pathway analyses to demonstrate that the metabolic profile of treated and control plants are very distinct and suggest the significant alteration of metabolic pathways by both *Acinetobacter* sp infection and metalaxyl treatment.

## References

[1] Borba N (2008). La papa: un alimento básico. Posibles impactos frente a la producción de la papa transgénica.. http://www.rapaluruguay.org/transgenicos/Papa/Papa.pdf.

[2] Alonso FA (2002). El cultivo de la patata.

[3] Thurston HD, Schultz O (1990). Late blight. Compendium of potato diseases. W.J. Hooker.

[4] Acuña IB (2008). Manejo integrado del tizón tardío y estrategias de control químico.. http://www.inia.cl/medios/biblioteca/informativos/NR35165.pdf.

[5] Pérez W, Forbes G (2008). Manual técnico: El tizón tardío de la papa.. http://www.scribd.com/doc/36688926/Manual-Tecnico-El-tizon-tardio-de-la-papa.

[6] Sukul P, Spiteller M (2000). Metalaxyl: persistence, degradation, metabolism, and analytical methods.. Rev Environ Contam Toxicol.

[7] World Health Organization and Food and Agriculture Organization of the United Nations (2004). Pesticides Residues in Food 2004. Evaluations Part I- Residues. FAO Plant Production and Protection Paper 182/1.. http://www.fao.org/ag/AGP/AGPP/Pesticid/JMPR/Download/2004_eva/JMPR2004eva.pdf.

[8] Food and Agriculture Organization of the United Nations (1995). Pesticides Residues in Food-1995: Evaluations Part 1-Residues, Part 1.. http://books.google.com/books?id=1Dl3YHLoqpEC&pg=PA512&dq=ApplicationofmetalaxylinBelgium&hl=en&ei=1cvNTonyBq3UiAKjzdDRCw&sa=X&oi=book_result&ct=result&resnum=2&ved=0CEAQ6AEwAQ#v=onepage&q=Application%20of%20metalaxyl%20in%20Belgium&f=false.

[9] Riveros FB, Sotomayor R, Rivera V, Secor G, Espinoza B (2003). Resistencia de *Phytophthora infestans*. (Montagne) de Bary A metalaxil en cultivo de papas en el norte de Chile.. Agricultura Técnica (Chile).

[10] Kamrin MA (1997). Pesticide profiles: toxicity, environmental impact, and fate.

[11] Businelli M, Patumi M, Marucchini C (1984). Identification and determination of some metalaxyl degradation products in lettuce and sunflower.. J Agric Food Chem.

[12] Gross D (1986). Uptake, translocation and metabolism of metalaxyl in higher plants..

[13] Owen WJ, Donzel B (1986). Oxidative degradation of chlortoluron, propiconazole and metalaxyl in suspension cultures of various crop plants.. Pestic Biochem Physiol.

[14] Cole DJ, Owen WJ (1987). Metabolism of metalaxyl in cell suspension cultures of *Lactuca sativa* L. and *Vitis vinifera* L.. Pestic Biochem Physiol.

[15] Zadra C, Marucchini C, Zazzerini A (2002). Behavior of metalaxyl and its pure R-enantiomer in sunflower.. J Agric Food Chem.

[16] McGuinness MC, Mazurkiewicz V, Brennan E, Dowling DN (2007). Dechlorination of pesticides by a specific bacterial glutathione S-transferase, Bphk^LB400^: potential for bioremediation.. Eng Life Sci.

[17] McGuinness M, Dowling D (2009). Plant-associated bacterial degradation of toxic organic compounds in soil.. Int J Environ Res Public Health.

[18] Germaine KJ, Liu X, Cabellos GG, Hogan JP, Ryan D (2006). Bacterial endophyte-enhanced phytoremediation of the organochlorine herbicide 2,4-dichlorophenoxyacetic acid.. FEMS Microbiol Ecol.

[19] Strobel G, Daisy B, Castillo U, Harper J (2004). Natural products from endophytic microorganism.. J Nat Prod.

[20] Rosenblueth M, Matinez-Romero E (2006). Bacterial endophytes and their interactions with hosts.. The American Phytophathology Society.

[21] Reinhold-Hurek B, Hurek T (2011). Living inside plants: bacterial endophytes.. Plant Biol.

[22] Ryan RP, Germaine K, Franks A, Ryan DJ, Dowling DN (2008). Bacterial endophytes: recent developments and applications.. FEMS Microb Lett.

[23] Bailey AM, Coffey MD (1986). Characterization of microorganisms involved in accelerated biodegradation of metalaxyl and metolachlor in soils.. Can J Microbiol.

[24] Zuno-Floriano FG, Estrada-de los Santos P, Gallegos-Infante JA, Rocha-Guzman NE, Aldana-Madrid ML (2009). Producción *in vitro* de plántula de papa inoculada con *Pseudomonas* sp.. Terra Latinoam.

[25] Pelczar M, Reid R, Chan ECS (1998). Microbiología.

[26] Massoud AH, Derbalah AS, Belal el-SB (2008). Microbial detoxification of metalaxyl in aquatic system.. J Environ Sci.

[27] BioRad (1994). Protein assay protocols.. http://www.bio-rad.com/webroot/web/pdf/lsr/literature/Bulletin_9004.pdf.

[28] Pelczar M, Chan ECS (1988).

[29] Grifoni A, Bazzicalupo M, Di Serio C, Fancelli S, Fani R (1995). Identification of Azospirillum strains by restriction fragment length polymorphism of the 16S rDNA and of the histidine operon.. FEMS Microbiol Lett.

[30] Sahay NS, Varma A (1999). *Piriformospora indica*: A new biological hardening tool for micropropagated plants.. FEMS Microbiol Lett.

[31] Barka EA, Belarbi A, Hachet C, Nowak J, Audran JC (2000). Enhancement of in vitro growth and resistance to gray mould of *Vitis vinifera* co-cultured with plant growth-promoting rhizobacteria.. FEMS Microbiol Lett.

[32] Martinez L, Caballero-Mellado J, Orozco J, Martinez-Romero E (2003). Diazotrophic bacteria associated with banana (*Musa* spp.). Plant Soil.

[33] Dini FA, de Araujo WL, de Azevedo JL, van Elsas JD, Nunes da Rocha U (2009). Endophytic colonization of Potato (*Solanum turerosum* L.) by a novel competent bacterial endophyte, *Pseudomonas putida* strain P9, and its effect on associated bacterial communities.. Appl Environ Microbiol.

[34] Anderote FD, Rocha UN, Araujo WL, Azevedo JL, van Overbeek LS (2010). Effect of bacterial inoculation, plant genotype and developmental stage on root-associated and endophytic bacterial communities in potato (*Solanum tuberosum*).. Antonie van Leeuwenhoek.

[35] Ma Y, Rajkumar M, Luo Y, Freitas H (2011). Inoculation on endophytic on host and non-host plants-Effects on plant growth and Ni uptake.. J Hazard Mater.

[36] Fiehn O, Wohlgemuth G, Scholz M, Kind T, Lee do Y (2008). Quality control for plant metabolomics: reporting MSI-compliant studies.. The Plant Journal.

[37] Tolstikov V (2011). Metabolic Biomarkers Discovery Project (MBDP) in Pancreatic Cancer Diagnostic Test Development.. http://www.selectbiosciences.com/conferences/MDWC2011/Agenda.aspx.

[38] Pooja S, Suri CR, Cameotra SS (2004). Isolation of a member of Acinetobacter species involved in atrazine degradation.. Biochem Biophys Res Commun.

[39] Liu FY, Hong MZ, Liu DM, Li YW, Show PS (2007). Biodegradation of methyl parathion by Acinetobacter radioresistens USTB-04.. J Environ Sci (China).

[40] Xie S, Liu J, Li L, Qiao C (2009). Biodegradation of malathion by Acinetobacter johnsonii MA19 and optimization of cometabolism substrates.. J Environ Sci (China).

[41] Hongsawat P, Vangnai AS (2011). Biodegradation pathways of chloroanilines by Acinetobacter baylyi strain GFJ2.. J Hazard Mater.

[42] Chanika E, Georgiadou D, Soueref E, Karas P, Karanasios E (2011). Isolation of soil bacteria able to hydrolyze both organophosphate and carbamate pesticides.. Bioresour Technol.

[43] Turnbull GA, Morgan JA, Whipps JM, Saunders JR (2001). The role of bacterial motility in the survival and spread of *Pseudomonas fluorescens* in soil and in the attachment and colonisation of wheat roots.. FEMS Microbiol Ecol.

[44] García-González MM, Farías-Rodríguez R, Peña-Cabriales JJ, Sánchez-Yáñez JM (2005). Inoculación del trigo var. pavón con *Azospirillum* spp. y *Azotobacter beijerinckii*.. Terra Latinoam.

[45] Prieto P, Mercado-Blanco J (2008). Endophytic colonization of olive roots by the biocontrol strain *Pseumonas fluorescens* PICF7.. FEMS Microbiol Ecol.

[46] Rainey PB (1999). Adaptation of Pseudomonas fluorescens to the plant rhizosphere.. Environ Microbiol.

[47] Araujo WL, Marcon J, Maccheroni W, van Elsas JD, van Vuurde JWL (2002). Diversity of endophytic bacterial populations and their interaction with *Xylella fastidiosa* in citrus plants.. Appl Environ Microbiol.

[48] Hansen M, Kragelund L, Nybroe O, Sørensen J (1997). Early colonization of barley roots by *Pseudomonas fluorescens* studied by immunofluorescence technique and confocal laser scanning microscopy.. FEMS Microbiol Ecol.

[49] Sturz AV, Nowak J (2000). Endophytic communities of rhizobacteria and the strategies required to create yield enhancing associations with crops.. Appl Soil Ecol.

[50] Germaine K, Keogh E, Garcia-Cabellos G, Borremans B, van der Lelie D (2004). Colonisation of poplar trees by *gfp* expressing bacterial endophytes.. FEMS Microbiol Ecol.

[51] Adachi K, Nakatani M, Mochida H (2002). Isolation of an endophytic diazotroph, Klebsiella oxytoca, from sweet potato stems in Japan.. J soil Sci Plant Nutr.

[52] Garbeva P, van Overbeek LS, van Vuurde JWL, van Elsas JD (2001). Analysis of endophytic bacterial communities of potato by plating and denaturing gradient gel electrophoresis (DGGE) of 16S rDNA based PCR fragments.. Microbiol Ecol.

[53] Elvira-Recuenco M, van Vuurde JW (2000). Natural incidence of endophytic bacteria in pea cultivars under field conditions.. Can J Microbiol.

[54] Chelius MK, Triplett EW (2000). Immunolocalization of dinitrogenase reductasa produced by *Klebsiella pneumonia* in association with *Zea mays* L.. Appl Environ Microbiol.

[55] Hartmann A, Stoffels M, Eckert B, Kirchhof G, Schloter M (2000). Analysis of the presence and diversity of diazotrophic endophytes..

[56] Verma SC, Singh A, Chowdhury SP, Tripathi AK (2004). Endophytic colonization ability of two deep-water rice endophytes, *Pantoea* sp. and *Ochrobactrum* sp. using green fluorescent protein reporter.. Biotechnol Lett.

[57] Torres-Rubio MG, Valencia-Plata SA, Bernal-Castillo J, Martinez-Nieto P (2000). Isolation of enterobacteria, *Azotobacter* sp. and *Pseudomonas* sp., producers of indole-3-acetic acid and siderophores, from Colombian rice rhizosphere.. Rev Lat-Amer Microbiol.

[58] Nelson LM (2004). Plant growth promoting rhizobacteria (PGPR): Prospects for new inoculants.. http://www.plantmanagementnetwork.org/sub/cm/review/2004/rhizobacteria/Nelson.pdf.

[59] Yanni YG, Rizk RY, El-Fattah FKA, Squartini A, Corich V (2001). The beneficial plant-growth promoting association of *Rhizobium leguminosarum* bv. Trifolii with rice roots.. Aust J Plant Physiol.

[60] Peña HB, Reyes I (2007). Aislamiento y evaluación de bacterias fijadoras de nitrógeno y disolventes de fosfatos en la promoción del crecimiento de la lechuga (*Lactuca sativa* L.).. Interciencia.

[61] Weyens N, van der Lelie D, Taghavi S, Vangronsveld J (2009). Phytoremediation: plant-endophyte partnership take the challenge.. J Plant Biotechnol.

[62] Claassens MMJ, Vreugdenhil D (2000). Is dormancy breaking of potato tubers the reverse of tuber initiation?. Potato Res.

[63] Holland MA (1997). Occam's razor applied to hormonology. Are cytokinins produced by plants?. Plant Physiol.

[64] Van Staden J, Davey JE (1979). The synthesis, transport and metabolism of endogenous cytokinin.. Plant Cell Environ.

[65] Visser RGF, Vreugdenhil D, Hendriks T, Jacobsen E (1994). Gene expression and carbohydrate content during stolon to tuber transition in potatoes (*Solanum tuberosum*).. Physiol Plant.

[66] Frommel MI, Nowak J, Lazarovits G (1993). Treatment of potato tubers with a growth promoting *Pseudomonas* sp.: Plant growth responses and bacterium distribution in the rhizosphere.. Plant Soil.

[67] Nowak J, Bensalim S, Smith CD, Dunbar C, Asiedu SK (1999). Behaviour of plant material issued from in vitro tuberization.. Potato Res.

[68] Sturz AV (1995). The role of endophytic bacteria during seed decay in potato tuberization.. Plant Soil.

[69] Mehta N, Saharan GS, Kathpal TS (1997). Absorption and degradation of metalaxyl in mustard plant (*Brassica juncea*).. Ecotoxicol Environ Saf.

[70] Badaway HMA, Mowafy MM, El-Megeed NMa, Kandil MA (2009). Persistence in tomato leaves of oxamyl and metalaxyl alone or in combination under different environmental conditions.. http://conf2009.agr.cu.edu.eg/volum1/12.pdf.

[71] Hamilton D (2004). METALAXYL-M (212).. http://www.fao.org/ag/AGP/AGPP/Pesticid/JMPR/Download/2004_eva/MetalaxylM.pdf.

[72] Roessner U, Wagner C, Kopka J, Trethewey RN, Willmitzer L (2000). Simultaneous analysis of metabolites in potato tuber by gas chromatography-mass spectrometry.. Plant J.

[73] Farre EM, Tiessen A, Roessner U, Geigenberger P, Trethewey RN (2001). Analysis of the compartmentation of glycolytic intermediates, nucleotides, sugars, organic acids, amino acids, and sugar alcohols in potato tubers using a nonaqueous fractionation method.. Plant Physiol.

[74] Defernez M, Gunning YM, Parr AJ, Shepherd LV, Davies HV (2004). NMR and HPLC profiling of potato with genetic modifications to metabolomic pathways.. J Agric Food Chem.

